# Invasive Bacterial Pathogens and their Antibiotic Susceptibility Patterns in Jimma University Specialized Hospital, Jimma, Southwest Ethiopia

**DOI:** 10.4314/ejhs.v21i1.69038

**Published:** 2011-03

**Authors:** Tizazu Zenebe, Subbaram Kannan, Daniel Yilma, Getenet Beyene

**Affiliations:** 1 Department of medical microbiology, Semera University; 2 Department of Laboratory Sciences and Pathology, College of Public Health and Medical Sciences, Jimma University; 3 Department of internal medicine, Jimma University Specialized Hospital, Jimma University

**Keywords:** invasive bacteria, antimicrobial resistance, Jimma, Ethiopia

## Abstract

**Background:**

Presence of microorganisms in the circulating blood whether continuously or intermittently is a threat to every organ in the body. Approximately 200,000 cases of bacteraemia occur annually with mortality rates ranging from 20–50%. Early diagnosis and appropriate treatment of these infections can make the difference between life and death. The aim of the present study was to determine the bacterial flora of the blood stream infections and their antibiotic susceptibility pattern.

**Methods:**

A cross sectional study was conducted on 260 adult febrile patients in Jimma University Specialized Hospital from 27 October 2009 to 26 March 2010. The positive blood cultures were examined and the organisms were identified as per standard procedures. Antimicrobial testing was performed for all isolates by disk diffusion techniques, according to *Clinical Laboratory Standards Institute* guide lines. The data was analyzed using SPSS for windows version 16 and Microsoft Office Excel.

**Results:**

From the total of two hundred sixty blood specimens only 23(8.8%) were positive to seven different types of bacteria. The isolated bacteria were: Coagulase negative staphylococci 6(26.1%), S. aureus 5 (21.7%), S. pyogens 3 (13.0%), *E. coli* 4(17.4%), K. pneumoniae 3(13.0%), Salmonella *spp.* 1(4.3%), and Citrobacter *spp.* 1(4.3%). The isolates showed high rates of resistance to most antibiotics tested. The range of resistance for gram positive bacteria were 0% to 85.7%, and for gram negative from 0% to 100%. None of the isolates were resistance to ciprofloxacin and ceftriaxone.

**Conclusion:**

Our study result showed the presence of invasive bacterial pathogens with high rate of resistance to most commonly used antibiotics used to treat bacterial infections. Therefore, timely investigation of bacterial flora of the blood stream infections and monitoring of their antibiotic resistance pattern plays an important role in reduction of the incidence of blood stream infections.

## Introduction

Bloodstream infection (BSI) remains one of the most important causes of morbidity and mortality through out the world. The disease may be short and self limiting or may result in death or serious morbidity including admission to intensive care or prolonged hospital stay (1). A wide range of bacteria has been described in febrile patients including gram negative bacteria such as *E. coli, P. aeruginosa, Klebsiella species Neisseria meningitidis, H. influenzae*, and gram positive such as *Coagulase negative staphylococci (CNS), S. aureus, S. pneumoniae, S. pyogens, S. agalactiae, and E. faecium*, (2,3,4,5,6).

Fever is a common presenting symptom to the emergency department and is attributable to a wide range of clinical diseases or infection (2, 7). The timely and appropriate use of antibiotics is currently the only way to treat bacteremia. However, many bacterial pathogens have become resistant to antibiotic regimens and become a serious public health concern with economic and social implications throughout the world. The infections caused by multi drug resistance organisms are more likely to prolong the hospital stay, increase the risk of death and require treatment with more expensive antibiotics (8).

In Ethiopia, except few reports on blood culture isolates from Tikur Anbessa Hospital (3, 9) data about the role and frequency of invasive bacteria on febrile adult cases and their susceptibility patterns is scarce. Thus providing updated information for local hospitals or regions for empirical choice of antibiotics in the treatment of bacteremia is very useful. Therefore, the present study was aimed at determining the frequency or occurrence of blood bacterial isolates in adult febrile cases and evaluate their drug susceptibility patterns at Jimma university specialized hospital.

## Materials and Methods

A prospective cross-sectional study was conducted in Jimma university specialized hospital (JUSH) Jimma, South west Ethiopia, to isolate invasive bacteria from adult febrile cases and assess their antibiotic susceptibility pattern from 27 October 2009 to 26 March 2010.

A total of 260 age ≥18 years old patients with fever (axillary temperature of ≥38°C) from the Out Patient Department were taken as study subject. The sample size was determined based on the prevalence rate of the study done by Asrat and Amanuel (3) and calculated with the formula recommended by Naing *et al.*, (10). Patients who were not voluntary to participate and took antibiotic within the last two weeks of visiting the hospital were excluded from this study.

After providing written informed consent, each patient was examined by physician and all relevant data (demographic, clinical, and laboratory data) were also recorded on questionnaire prepared for this purpose.

About 5 ml of venous blood was collected aseptically using blood sample collection sets and antiseptics (70% alcohol and 2% tincture iodine) and transferred into a bottle containing sterile Brain heart infusion (BHI) broth (Oxoid Ltd, UK), with 0.025% Sodium Polyanethol Sulfonate (SPS) [Himedia, India]. A minimum blood-to-broth ratio of 1 in 10 was maintained. Blood culture broths were incubated at 370C and checked for sign of bacterial growth daily up to 7 days, and bottles which showed signs of growth were further processed for gram stain and sub-cultured was made onto blood agar (Oxoid Ltd, UK), MacConkey agar (Oxoid Ltd, UK), Manitol salt agar (Oxoid Ltd, UK) and incubated at 37°C for 24 hours (11).

Blood culture broth with no bacterial growth after 7 days were sub-cultured before being reported as a negative result. The isolates were identified with colony morphology, gram staining reaction, biochemical and serological tests (11). Antimicrobial susceptibility testing was performed for the isolated organisms according to Clinical Laboratory Standards Institute (CLSI) guide lines (12, 13). The antibiotic discs and their concentrations were: Penicillin G (P, 10IU), Amoxicillin (AML, 20µg), Ampicillin (AMP, 10µg), Cephalothin (KF, 30µg) Tetracycline (TE, 30µg), Chloramphenicol (C, 30µg), Ciprofloxacin (CIP, 5µg), Trimethoprim+Sulphamethazole (SXT, 25µg) Gentamicin(CN, 10µg), Ceftriaxone (CRO, 30µg), Erythromycin (E, 10µg), Methicillin (MET, 5µg) and Nalidixic acid (NA, 30µg). All the antimicrobials used for the study were obtained from Oxoid, UK. The criteria used to select the antimicrobial agents tested were based on the availability and frequency of prescription for the management of bacterial infections in Ethiopia. The reference strains used as control were *E. coli* (ATCC 25922), and *S. aureus* (ATCC 25923). In this study multi-drug resistance was defined as simultaneous resistance to two or more antimicrobial agents.

Both the clinical and laboratory data were entered into a computer and analyzed using SPSS for widows version 16.0 and Microsoft Office Excel. Two-sided P values were calculated using the chi-square test or Fisher's exact test for some variables.

Ethical clearance from Jimma University Ethical Review Board and patient's consent was obtained with their signature after getting enough information on the purpose of the study.

## Results

Out of 260 patients 106 (40.8%) were males and 154 (59.2%) females. Their age ranged from 18–61 with median of 26 years. The majority of the patients 158 (60.7%) were between the age of 18 and 29 years. One hundred eighty seven (51.9%) patients were rural and 73 (48.1%) town residents.

**Figure 1:**
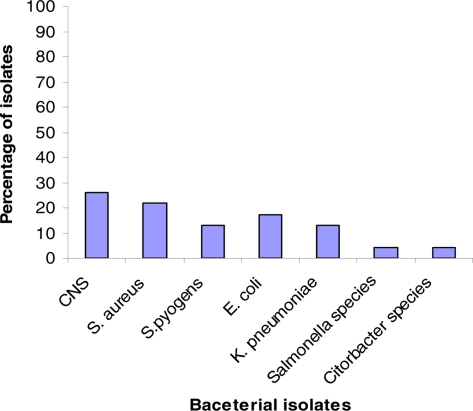
Frequency of blood bacterial isolates from adult febrile cases in Jimma University

From the 260 blood cultures a total of 23 bacterial strains were isolated from 23 different cases, with a culture positivity rate of 8.8% (Fig. 1). The overall rate of isolation were 60.9% (n= 14) for gram positive bacteria and 39.1% (n= 9) for gram negative bacteria. As it is shown on Fig 1, the predominant isolates were coagulase negative staphylococci (CNS) 6 (26.1%) followed by *S. aureus* 5 (21.7%) and the least were *Salmonella* species 1(4.3%), and *Citrobacter species* (4.3%).

Among the gram positive bacteria high resistance was observed to Penicillin (85.7%) and Ampicillin (71.4%) (Table 1). The gram negative bacteria showed 100% resistance against Ampicilin, 88.9% to Tetracycline, 77.9% to Chloroamphenicol, 77.8% to Co-trimoxazole, and 66.7% against Gentamicin (Table 2). Overall the range of resistance for gram positives was from 0% to 85.7%, and for gram negative from 0% to 100%. However, all bacterial isolates were sensitive to Ceftriaxone and Ciprofloxacin.

**Table 1:** Antibiotic resistance of gram positive bacterial isolates from blood culture in Jimma University Specialized Hospital Jimma town, Southwest Ethiopia, October, 2009 to March, 2010.

Name of isolates	Number of strains (%) resistance to											
	P	AMX	AM	C	CF	CIP	GM	CRO	TE	E	MET	SXT
***CNS* (n=6)**	4(66.7)	1(16.7)	2(33.3)	2(33.3)	0(0)	0(0)	1(16.7)	0(0)	1(16.7)	0(0)	2(33.3)	1(16.7)
***S. aureus(n=5)***	5(100.0)	2(40.0)	5(100.0)	5(100.0)	1(20.0)	0(0)	3(60.0)	0(0)	5(100.0)	2(40.0)	5(100.0)	3(60.0)
***S. pyogens* (n=3)**	3(100.0)	0(0)	3(100.0)	0(0)	0(0)	0(0)	0(0)	0(0)	1(33.3)	0(0)	0(0)	0(0)
***Total (n=14)***	12(85.7)	3(21.4)	10(71.4)	7(50)	1(7.1)	0(0)	4(28.6)	0(0)	7(50)	2(14.3)	7(50)	11(78.6)


**Key:**- P=Penicillin G, AMX=Amoxicillin, AM =Ampicillin, E= Erythromycin, CF =Cephalothin, TE=Tetracycline, C=Chloramphenicol, CIP=Ciprofloxacin, SXT= Trimethoprim+ Sulphamethazole, GM= Gentamicin, CRO= Ceftriaxone, MET= Methicillin, CNS= Coagulase negative staphylococcus

**Table 2:** Antibiotic resistance of gram negative bacterial isolates from blood culture Jimma University Specialized Hospital, Southwest Ethiopia from October 2009 to March 2010

Name of isolates	Number of strains (%) resistance to									
	AMX	AM	C	CF	CIP	GM	CRO	TE	NA	SXT
***E. coli* (n=4)**	0(0)	4(100)	4(100)	0(0)	0(0)	3(75)	0(0)	3(75)	0(0)	4(100)
***K. pneumoniae* (n=3)**	1(33)	3(100)	1(33)	1(33)	0(0)	(33)	(0)	3(100)	3(100)	3(100)
***Salmonella spp.* (n=1)**	1(100)	1(100)	1(100)	1(100)	0(0)	1(100)	0(0)	1(100)	0(0)	NT
***Citrobacter spp.* (n=1)**	1(100)	1(100)	1(100)	1(100)	0(0)	1(100)	0(0)	1(100)	0(0)	1(100)
***Total* (n=9)**	3(33)	9(100)	7(77.8)	3(33)	0(0)	6(66.7)	0(0)	8(88.9)	3(33)	8(88.9)


**Key:** - AMX =Amoxicillin NA=Nalidixic acid, AM =Ampicillin, CF =Cephalothin, TE= Tetracycline, C= Chloramphenicol, CIP= Ciprofloxacin, SXT= Trimethoprim+Sulphamethazole, GM= Gentamicin CRO= Ceftriaxone , MET= Methicillin

Most gram positive and gram negative isolates were resistant to more than three antibiotics. Over all five distinct antibiogram (resistance pattern) were observed in CNS-four in *S. aureus*, two in *S. pyogenes* and the antibiogram of gram negative bacteria were two in *E. coli* and two in *K. pneumonae* (Table 3). The resistance pattern of CNS varied from 3–6 drugs. Five of the total CNS isolates were multi drug resistant (resistant to 3 or more drugs). All the *S. aureus* isolates were multi drug resistant varying from 5–10 drugs whereas two and one S. *pyogenes* isolates were resistant to two and three drugs, respectively. All the gram negative bacteria isolates were multidrug resistant. The single isolate of *Salmonella spp.* and *Citrobacter spp.* were resistant to six and seven drugs, respectively.

**Table 3:** Resistance antibiogram pattern of the blood bacterial isolates Jimma University Specialized Hospital, Southwest Ethiopia from October 2009 – March 2010.

Blood isolates	Resistance antibiogram	No
Gram positive		
CNS	P,AM,C P,AM,MET P,C,TE P,AM,GM P,AM,C,CF,MET,SXT	1 1 1 1 1
S. aureus	P,AM,C,TE,MET P,AM,C,GM,TE,MET,SXT P, AMX,AM,C,GM,TE,E,MET,SXT P,AMX,AM,C,CF,GM,TE,E,ME,SXT	2 1 1 1
S. pyogens	P,AM P,AM,TE	2 1
Gram negative		
E. coli	AM,C,GM,SXT AM,C,GM,TE,SXT	1 3
K. pneumonae	AMX,AM,TE,NA AM, C, CF,GM,TE,SXT,NA	1 2
Salmonella spp.	AMX,AM,C,CF,TE,SXT	1
Citrobacter spp.	AMX,AM,C,CF,GM,TE,SXT	1


Key: Penicillin G = P, AMX =Amoxicillin NA=Nalidixic acid, AM =Ampicillin, E= Erythromycin, KF =Cephalothin, TE= Tetracycline, C= Chloramphenicol, SXT= Trimethoprim+Sulphamethazole, CN= Gentamicin, MET= Methicillin, CNS= Coagulase negative staphylococcus

## Discussion

Blood stream infections cause significant morbidity and mortality worldwide and are among the most common healthcare-associated infections. The isolated bacteria are numerous and their associated disease need urgent and invasive management with antimicrobial drugs (1).

The isolation rate (8.8%) in this study is lower compared to other studies reported in Addis Ababa hospital (21.4%) (3) and in another African and Asian countries like Malawi 17.6% (14), Nepal 23.1% (15), 34% in Gambia (16) and 40% in India (17), respectively. However, there are reports from other parts of the world that agree with the present findings such as in Nepal 10 % (19) and in India 9.94% (6). In the present study gram positive bacteria were the most leading isolates (60.9%) as compared to gram negative (39.1%) which is comparable with previous study done in Addis Ababa, where 62.6% of the isolates were gram positive and 37.4% gram negative, respectively (7). But different from the study results done in Nepal where 89.19% of the total isolates were gram negative and 10.81% gram positive (18). The possible explanation for the difference could be the difference in blood culture system, the study design, geographical location, nature of patient population, epidemiological difference of the etiological agents, and seasonal variation.

Among blood isolates CNS (26.1%) was the most common organism, followed by S. aureus (21.7%) and E. coli (17.4%) and similar findings have been reported in other studies (4, 5, 19). CNSs have been long regarded as non pathogenic but their important role as pathogens and their increasing incidence in different infection like bacteremia related to indwelling devices, central nervous systems shunt infections, native or prosthetic valve endocardiatis, urinary tract infections had been reported and it is also an important cause of morbidity and mortality in immunocopromised individuals (20). Other investigators have also reported an increase of true bacteremia by CNS in recent years (5). In the present study the second predominant isolates were *S. aureus*, 21.7%, which contrast to the study conducted in USA where the prevalence was 2.6% (21) but comparable with the study results done in Iowa, USA 27.4% of *S. aureus* bacteremia (22). *S. pyogenes* accounted 13.0% in the present study which is higher than reported by Usha Arora and Pushpa Devi, 1.24% (23). In this study, E. coli was accounted for 17.4% which agreed with study done in Australia (19%) (24) and higher than other studies (3, 4, 5, 19). *K. pneumoniae* accounted for 13.0% of the total blood isolates which is almost similar with study results in India done at different times (6, 19). The isolation rate of Salmonella species was 4.3% and comparable with the study done in Addis Ababa, 3.8% (3) and in Jimma, 5.3% (25).

The rate of resistance for the isolates to antibiotics used is comparatively higher than from the previous reports in Addis Ababa (3, 9) but in agreement with a study done by other investigator elsewhere in the world (19). Overall the range of resistance for gram positives was from 0% to 85.7%, and for gram negative from 0% to 100% which is different from the study result done in Addis Ababa where the rates for gram positive bacteria ranged from 12% to 76%, and for gram negatives ranged from 8% to 46%(3). The increased resistant blood isolates in this study may be an indication of indiscriminate and continuous use of sub-therapeutic doses of commonly available antimicrobials both in the veterinary and public health sectors. Amongst the gram positive organism's high resistance was observed with Ampicillin (71.4%) which is consistent with other study, where the rate of resistance was 74.61 % (19). Further more, high resistance among gram negatives was seen against Ampicillin (82.6%), Tetracycline (65.2%) and Chloroamphenicol (60.9%) which is in agreement with the study findings by Usha Arora and Pushpa Devi in India (19). Almost all isolates were resistant to two or more antimicrobials. All *S. aureus* were multidrug resistant which is higher than previous study in Jimma (72.9%) (26). All *S. aureus* isolates were resistant for Penicillin, Ampicillin, chlorampphenicol, Tetracycline and Methicillin. This is in contrast with the study result of Balta and Deribie, where resistance rate against Ampicillin was 87.1%, 70.6 % to chlorampphenicol, and 51.8% against Methicillin (26). The single isolate of *Salmonella* species and *Citrobacter* species were found to be resistant to six or more antimicrobials. The high resistance rate of *Salmonella* species is similar to the study result of Beyene et al where most of the Salmonella isolates were multidrug resistant (25). However, among the antibiotics used for susceptibility testing ceftriaxone and ciprofloxacin were effective against all the isolates.

In Ethiopia the unregulated over-the-counter sale of these antimicrobials, mainly for self-treatment of suspected infection in humans, and to a lesser extent for use in animals without prescription, would inevitably lead to emergence and rapid dissemination of resistance. In addition, availability of cheaper generic drugs of variable quality in the market for treatment of bacterial infections may also contribute to the increased level of resistance. A study on practice of self-medication in Jimma town showed that out of the 152 ill people, 27.6% were self-medicated (27). The relative lesser cost (35.7%) was the major reason for using self medication.

In conclusion, our study result showed the presence of invasive bacterial pathogens with high rate of resistance to most commonly used antibiotics to treat bacterial infections. Therefore, timely investigation of bacterial flora of the blood stream infections and monitoring of their antibiotic resistance pattern plays an important role in reduction of the incidence of blood stream infections.
